# Energy finance strategy and governance nexus with economic growth: Results from emerging economies

**DOI:** 10.1371/journal.pone.0314286

**Published:** 2024-12-05

**Authors:** Md. Mominur Rahman, Fataraz Zahan, Md. Farijul Islam

**Affiliations:** 1 Bangladesh Institute of Governance and Management, University of Dhaka, Dhaka, Bangladesh; 2 Department of Marketing, Jagannath University, Dhaka, Bangladesh; China University of Mining and Technology - Beijing, CHINA

## Abstract

The rapid economic development in emerging economies, particularly in BRICS nations, is closely intertwined with their energy consumption and financial investment in energy sectors. However, the global shift towards sustainability has raised concerns about the continued reliance on fossil fuels and the environmental implications of such practices. Energy finance—particularly the balance between fossil fuel energy finance (FFEF) and renewable energy finance (RENF)—plays a critical role in shaping economic growth trajectories in these economies. At the same time, governance frameworks can either enhance or hinder the effectiveness of energy finance strategies. As the global push for sustainability intensifies, the need to balance these two energy sources becomes increasingly important. This study investigates the impact of energy finance on economic growth in BRICS nations and explores how governance moderates these relationships. Using data from the World Development Indicators (2000–2023) and employing econometric models, including Fully Modified Ordinary Least Squares (FMOLS) and Dynamic Ordinary Least Squares (DOLS) for robustness, the findings reveal that both FFEF and RENF positively impact EGR. However, RENF also offers the added benefit of environmental sustainability, positioning it as a viable alternative for economic development. Good governance emerges as a critical factor that can mitigate the negative environmental effects of FFEF and further amplify the positive impact of RENF on EGR. The study highlights that BRICS nations have the option to shift from FFEF to RENF, as RENF not only promotes economic growth but also aligns with environmental goals. Strengthening governance frameworks will be essential in facilitating this transition and supporting sustainable economic growth.

## Introduction

Energy finance, specifically the financing of fossil fuel and renewable energy sources, has long been a crucial component in the economic development of both advanced and emerging economies [1, 2]. Historically, FFEF has been the dominant driver of industrialization and economic growth, particularly since the 19th century [3]. Fossil fuels—such as coal, oil, and natural gas—have powered industries, transportation, and energy infrastructure globally, facilitating rapid economic expansion [4]. This pattern is especially evident in emerging economies like the BRICS nations (Brazil, Russia, India, China, and South Africa), which have relied heavily on fossil fuels to meet their increasing energy demands [5–9]. As these nations industrialized, fossil fuel consumption skyrocketed, solidifying FFEF as a central pillar of economic strategy [9–11].

However, the historical reliance on fossil fuels has also created challenges, particularly in terms of environmental sustainability. The global shift toward renewable energy in the 21st century reflects growing concerns over climate change, greenhouse gas emissions, and the finite nature of fossil fuel reserves [12–14]. RENF emerged as an alternative, with countries worldwide seeking to balance economic growth with environmental sustainability. In recent decades, BRICS nations have begun to explore renewable energy sources, but the transition remains uneven [13, 15, 16]. As a result, energy finance in these economies now exists in a state of flux, caught between the need to sustain economic growth and the imperative to reduce environmental harm.

Despite the historical dominance of FFEF in BRICS economies, increasing awareness of environmental degradation and climate risks has sparked a growing interest in RENF as a sustainable alternative [1, 6, 17]. However, transitioning from fossil fuels to renewable energy is not a simple process, particularly in emerging economies that rely heavily on fossil fuel industries for economic stability and growth. The core issue is understanding how energy finance, in its various forms, influences economic growth trajectories in BRICS nations and how governance structures can shape this relationship [18]. Although both FFEF and RENF can drive economic growth, their environmental implications differ significantly [19]. Therefore, it is crucial to investigate how governance—defined here as the systems, policies, and institutions that regulate economic and environmental practices—affects the balance between these two energy finance strategies. The issue is significant for several reasons. First, BRICS nations are major players in the global economy, collectively representing over 40% of the world’s population and a significant share of global GDP [8, 20]. Their energy strategies, especially concerning fossil fuels and renewable energy, will significantly impact global economic and environmental outcomes [7, 11]. If BRICS nations can successfully transition to renewable energy without compromising economic growth, they could serve as a model for other emerging economies [5, 21]. Conversely, continued reliance on fossil fuels could exacerbate global environmental problems, particularly climate change [7, 19, 22]. Second, the governance aspect of energy finance is critical yet underexplored. While many studies have examined the economic and environmental impacts of FFEF and RENF, few have looked at how governance moderates these effects. Understanding the role of governance is essential because effective governance can amplify the benefits of renewable energy and mitigate the harmful effects of fossil fuels. Poor governance, on the other hand, may hinder the transition to sustainable energy, perpetuating the economic and environmental risks associated with fossil fuel reliance [15, 23]. Finally, the significance of this issue extends beyond economics and environmental science. It touches on global efforts to combat climate change, reduce inequality, and promote sustainable development. By exploring the intersection of energy finance, governance, and economic growth in BRICS nations, this study addresses a critical gap in the literature and provides insights that could inform global energy policies.

Governance theory is highly relevant to this study, as it provides the framework for understanding how various political, economic, and institutional factors influence energy finance strategies [24, 25]. Governance encompasses the policies, regulations, and institutional structures that shape decision-making processes and the implementation of energy finance [26, 27]. Good governance refers to the presence of transparent, accountable, and effective institutions that can facilitate the transition from FFEF to RENF while ensuring sustained economic growth. Governance theory posits that the quality of governance can significantly affect the outcomes of public policy, particularly in complex domains like energy finance [18, 28, 29]. In countries with strong governance frameworks, policies that promote renewable energy finance are more likely to be successfully implemented, leading to positive economic and environmental outcomes. In contrast, countries with weaker governance may struggle to manage the transition away from fossil fuels, facing challenges such as corruption, policy inconsistency, and regulatory inefficiencies [30, 31]. Thus, governance theory helps to explain why similar energy finance strategies can have different outcomes in different countries and why governance plays a moderating role in the relationship between energy finance and economic growth.

While there is substantial literature on the economic and environmental effects of energy finance [3, 5, 12, 13, 15, 16, 19, 23, 32], gaps remain, particularly concerning the role of governance in shaping these effects in emerging economies [19, 26, 29]. Furthermore, there is limited understanding of how governance structures moderate these effects, especially in emerging economies with varying degrees of institutional strength and political stability [33–36]. Existing research treats governance as a secondary variable rather than a central factor influencing the success of energy finance strategies [19, 29, 30]. This study aims to address this gap by examining governance as a key moderating factor in the relationship between energy finance and economic growth. The study seeks to answer the following key questions:

1. How does fossil fuel energy finance affect economic growth in BRICS nations?
2. How does renewable energy finance impact economic growth in BRICS nations?
3. What role does governance play in moderating the effects of FFEF and RENF on economic growth?

This study makes important contributions to the field of energy finance and sustainable development. First, it fills a gap in the literature by examining the simultaneous effects of FFEF and RENF on economic growth in BRICS nations, an area that has received limited attention in previous research [1, 15, 17, 19, 37]. Second, it advances governance theory by exploring how governance moderates the relationship between energy finance and economic growth, offering new insights into the role of governance in promoting sustainable development. Third, the study provides empirical evidence on the effectiveness of governance frameworks in shaping the transition from fossil fuels to renewable energy. By focusing on BRICS nations, the research adds a new dimension to the study of energy finance, highlighting the unique challenges and opportunities faced by emerging economies.

The rest of the paper includes: literature review section, data and methods section, results section, discussions section, and conclusions section.

## Literature review

### Theoretical underpinning

Governance theory provides a vital framework for understanding how institutions, policies, and regulatory mechanisms shape economic outcomes, particularly in relation to energy finance and economic growth [25, 27]. At its core, governance theory emphasizes the importance of institutional structures, transparency, accountability, and rule of law in ensuring the efficient allocation of resources and fostering sustainable economic development [19, 35]. In the context of energy finance, governance theory posits that well-functioning institutions and regulatory frameworks can significantly influence how financial resources are allocated to both FFEF and RENF. Good governance ensures that these financial flows are directed toward projects that not only maximize economic growth but also minimize environmental harm and promote long-term sustainability [15, 29, 30]. Conversely, weak governance, characterized by corruption, lack of transparency, and regulatory inefficiencies, can lead to mismanagement of resources, undermining both economic growth and environmental goals [25, 32, 33].

In the context of BRICS nations, governance theory becomes particularly relevant, as these emerging economies often face governance challenges while seeking rapid economic growth. Effective governance frameworks in these countries can serve as critical moderators in the relationship between energy finance and economic growth [26, 36]. Governance theory suggests that when regulatory institutions function effectively, they can mitigate the negative effects of FFEF, such as environmental degradation, and amplify the positive impacts of RENF on economic growth. Additionally, governance theory highlights the role of government accountability and institutional transparency in fostering investor confidence, which is essential for attracting long-term investments in both energy sectors [15, 29]. By applying governance theory to the energy finance and economic growth nexus, this study underscores the importance of governance in shaping the sustainability of economic development in BRICS nations, offering insights into how improved governance structures can balance economic objectives with environmental imperatives [27, 35].

### Fossil fuel energy finance and economic growth

Fossil fuel energy finance has historically been a primary driver of economic growth, particularly in emerging economies where industrialization, infrastructure development, and urbanization rely heavily on fossil fuel consumption [3, 4]. The availability and investment in fossil fuel resources such as coal, oil, and natural gas have powered industries, created jobs, and driven technological advancements, ultimately boosting national incomes and overall economic growth [22, 38]. Investment in fossil fuel infrastructure leads to immediate and substantial economic benefits. For example, building refineries, pipelines, and transportation networks generates employment, stimulates local industries, and drives both domestic and export-oriented growth [2, 6]. In BRICS nations, fossil fuel energy continues to be a major contributor to GDP growth, providing affordable energy that supports manufacturing, agriculture, and service sectors [8, 38]. The affordability and availability of fossil fuels also ensure energy security, which is crucial for maintaining consistent economic growth.

The environmental degradation caused by fossil fuel extraction, production, and consumption—such as air pollution, greenhouse gas emissions, and climate change—poses significant long-term risks to sustainable economic development [5, 39, 40]. Fossil fuels are also a finite resource, meaning that over-reliance on them can lead to economic vulnerability as reserves deplete or global energy markets shift toward renewable sources. Furthermore, the costs of mitigating environmental damage, such as health-related expenses and climate adaptation, can burden economies, offsetting the initial economic gains [19, 37, 41]. Over time, continued investment in fossil fuels may undermine economic growth due to the rising costs associated with environmental damage and global shifts toward cleaner energy alternatives.

Energy-related research and development reveals those improvements in energy storage, renewable energy, and energy efficiency. Environmental and social concerns, like community development, air and water quality improvements, and mitigating the effects of climate change, are being taken into account more and more in climate finance [17, 42, 43]. Long-term economic growth can be stimulated by investments in sustainable practices and cleaner energy technology, which can reduce environmental risks and increase resource efficiency while promoting social stability [14, 26, 44]. These developments may stimulate economic growth and competitiveness by lowering costs, increasing productivity, and spawning new businesses [39, 45, 46].

Different studies fundings promotes growth by lowering costs, increasing energy efficiency, and assisting with sustainable development, in contrast conventional finance supports the economy as a whole by providing cash for all sectors [9, 16, 22, 32]. For instance, the development, installation, and upkeep of solar power infrastructure has generated millions of employments in India. Their investments in renewable energy have drawn interest from both local and foreign sources. These investments have boosted industrial growth, decreased dependency on costly energy imports, and created job opportunities [40]. Given these mixed effects, the study aims to explore the extent to which FFEF continues to drive economic growth in BRICS nations while considering the potential risks of over-reliance on fossil fuels. This leads to the following hypothesis:

*H1*: *Fossil fuel energy finance boosts economic growth in BRICS nations*.

### Renewable energy finance and economic growth

Renewable energy finance has emerged as a sustainable alternative to fossil fuels, offering both economic and environmental benefits [1, 32]. Investments in renewable energy technologies such as solar, wind, hydro, and bioenergy are becoming increasingly attractive to policymakers and investors, particularly in the context of growing concerns about climate change and environmental degradation. RENF can contribute to economic growth by creating new industries, stimulating innovation, and reducing dependence on volatile fossil fuel markets [7, 8]. Renewable energy investments create high-skilled jobs in sectors such as engineering, manufacturing, and research and development. As economies shift toward cleaner energy, new industries emerge, fostering entrepreneurship and innovation. In BRICS nations, the potential for renewable energy development is vast due to their diverse geographical landscapes, which allow for the harnessing of solar, wind, and hydropower on a large scale [5, 26, 47]. Furthermore, by reducing reliance on imported fossil fuels, RENF can improve trade balances and enhance energy security, leading to more stable and sustained economic growth.

Renewable energy is a cleaner and more sustainable option, meaning that the long-term costs associated with environmental degradation are significantly lower compared to fossil fuels [6, 18, 19, 38]. This positions renewable energy as a driver of not only economic growth but also sustainable development. As global energy markets increasingly favor low-carbon technologies, economies that prioritize RENF are likely to experience more sustainable, resilient growth. But the initial costs of transitioning to renewable energy can be high, requiring substantial public and private investment [22, 40]. In some cases, the economic benefits of RENF may take time to materialize due to the long development cycles of large-scale renewable energy projects. Additionally, if not properly managed, the renewable energy sector can face barriers such as regulatory challenges, technological bottlenecks, and market integration issues, which can slow economic growth [6, 9, 18, 38].

The impact of renewable energy consumption and economic growth in emerging economies was studied by Nguyen et al. [13]. They discovered that the consumption of renewable energy and growth are correlated in both directions, suggesting that economic expansion drives up energy investments and, on the other hand, that energy investments encourage expansion through higher productivity. Apergis and Payne [48] discovered a favourable correlation between the consumption of renewable energy and economic growth through a panel study involving multiple countries. According to their theory, this happens as a result of investments in renewable energy since they lessen the negative externalities of fossil fuels, such as pollution and fuel price volatility, which in turn fosters economic stability.

Energy finance will play an even more crucial role in sustaining long-term economic growth as the world economy shifts towards sustainability by encouraging innovation, generating jobs, improving energy security, and promoting environmental sustainability. Renewable energy finance is a significant driver of economic development in these emerging economies [6, 13, 15]. In addition, China’s leadership in the production of renewable energy technologies has made it a major exporter of wind turbines and solar panels, further boosting economic growth, generating jobs, improving energy security, drawing in foreign investment, and promoting environmental sustainability [49]. Nonetheless, the long-term benefits of renewable energy finance—both in terms of economic growth and environmental sustainability—are clear, especially for nations like those in the BRICS bloc, which are poised to reap significant rewards from embracing cleaner energy solutions. Hence, the following hypothesis is proposed:

*H2*: *Renewable energy finance enhances economic growth in BRICS nations*.

### Good governance and economic growth

Good governance, characterized by transparency, accountability, rule of law, and effective institutions, plays a critical role in facilitating economic growth [25, 27, 30]. Governance influences economic growth by creating an environment conducive to investment, ensuring the efficient allocation of resources, and minimizing corruption and bureaucratic inefficiencies [19, 35]. Strong governance frameworks help build investor confidence by ensuring regulatory stability and the rule of law, which are essential for attracting long-term investments in energy infrastructure. In the context of BRICS nations, where public and private sector partnerships are crucial for large-scale energy projects, governance ensures that funds are allocated efficiently and that energy projects meet both economic and environmental goals [18, 26, 27]. Conversely, poor governance can hinder economic growth by creating an environment of uncertainty and inefficiency. Corruption, bureaucratic delays, and lack of regulatory transparency can discourage investment in both fossil fuel and renewable energy sectors [19, 30, 35]. In BRICS nations, where governance structures vary widely, poor governance can exacerbate the risks associated with energy finance, such as project delays, cost overruns, and environmental degradation.

Nevertheless, reducing the likelihood of corruption and mismanagement, good governance guarantees accountability and openness in the management of energy finance [7]. More investment in energy projects is attracted by transparent procedures for contract awarding and fund allocation, which boosts investor trust and accelerates economic growth [20]. Energy investments are stable and predictable when there is a well-run regulatory framework in place. Investment in energy projects is encouraged by regulations that are clear and uniform, as they lessen investor uncertainty. A climate that is favourable for drawing in capital, igniting the economy, and fostering sustainable growth in the energy sector is created by this stability [42].

By ensuring the advantages of energy finance are dispersed fairly throughout society, good governance fosters inclusive development [18]. To mitigate socioeconomic disparities and guarantee that marginalised groups reap the benefits of energy investments, policies that encourage social inclusion, community engagement, and stakeholder participation in energy decision-making processes can be instrumental in fostering economic growth on a wider scale [8]. However, openness, regulatory stability, effective resource allocation, risk management, and equitable development are all facilitated by strong governance, which increases the efficiency. Good governance can unleash the energy sector’s full potential as a catalyst for wealth and economic development by fostering an environment that is supportive of energy investments [7, 12].

Over the last two decades, the BRICS countries have seen tremendous economic growth, despite significant differences in the efficacy and transparency of their governance structures. The comprehension of the durability of progress in these countries hinges on the correlation between economic growth and sound governance [19]. Brazil has had slowed economic growth in recent years as a result of problems with governance, such as pervasive corruption and improper use of public funds. The nation has had times of rapid growth, particularly between 2000 and 2010, but poor administration has made long-term, sustainable growth more difficult [50]. Russia does not score well on global governance metrics, particularly when it comes to political freedom and openness, despite having a strong central authority [36, 51]. Given the pivotal role governance plays in shaping economic growth, the study emphasizes its importance in ensuring that energy finance strategies are effective and sustainable. The following hypothesis is proposed:

*H3*: *Good governance improves economic growth in BRICS economies*.

### Moderating effects of governance

Governance can strengthen or weaken the impact of energy finance on economic growth, depending on the quality of governance structures in place [18, 27, 28]. In nations with strong governance frameworks, both FFEF and RENF are likely to have more positive effects on economic growth. Good governance ensures that fossil fuel projects are managed in ways that mitigate environmental harm while renewable energy projects are implemented efficiently and sustainably. Strong governance frameworks can mitigate the environmental risks associated with fossil fuels by enforcing regulations on emissions and waste management [33, 35]. Similarly, good governance can ensure that renewable energy projects are not only economically viable but also aligned with long-term sustainability goals [26, 27]. This strengthens the positive relationship between energy finance and economic growth, ensuring that investments lead to both economic prosperity and environmental sustainability. In contrast, weak governance can exacerbate the negative effects of FFEF and undermine the benefits of RENF. Poor regulatory oversight can lead to environmental degradation, corruption, and inefficiency, all of which reduce the overall economic benefits of energy finance [15, 30]. In BRICS nations, where governance structures are sometimes inconsistent or underdeveloped, weak governance can diminish the potential of both FFEF and RENF to drive economic growth. Given these potential moderating effects, the study aims to explore how governance shapes the relationship between energy finance and economic growth, leading to the following hypothesis:

*H4*: *Good governance strengthens the relationship between energy finance and economic growth in BRICS nations*.

## Data and methods

### Data and variables

The data sample selected for emerging countries includes the original BRICS members: Brazil, Russia, India, China, and South Africa. Other BRICS nations, such as those that joined the expanded BRICS group recently, are not included in this analysis due to the lack of consistent and comprehensive data over the entire study period from 2000 to 2023. Additionally, the original BRICS countries provide a robust dataset that aligns with the study’s focus on long-term economic and energy finance trends [5].

BRICS countries are chosen for this study due to their significant roles as major emerging economies with diverse energy profiles and governance structures [5, 10, 52]. These countries collectively represent a substantial portion of the world’s population, GDP, and energy consumption, making them ideal for examining the interplay between energy finance, governance, and economic growth [10]. Furthermore, the economic development trajectories and policy environments in BRICS nations offer valuable insights into how different governance quality levels can influence the impact of energy finance on economic growth.

The data covers the period from 2000 to 2023 and is sourced from the World Development Indicators (WDI) database. Table 1 illustrates the data description used in this study. The WDI provides reliable and comprehensive statistical data essential for empirical analysis, ensuring consistency with the existing literature referenced in this paper.

**Table 1:** Data and description.

Sign	Definition	Measure	Source
EGR	Economic Growth	GDP growth (annual %)	WDI
FFEF	Fossil Fuel Energy Finance	Fossil fuel energy finance (% of total)	WDI
RENF	Renewable Energy Finance	Renewable energy finance (% of total final energy consumption)	WDI
GGOV	Good Governance	Government Effectiveness: Percentile Rank	WDI
GGOV*FFEF	Moderator of EGR and FFEF	MFFE is the moderator of economic growth and fossil fuel energy finance. It is calculated by GGOV*FFEF.	Authors’ Calculation
GGOV*RENF	Moderator of EGR and RENF	MREF is the moderator of economic growth and renewable energy finance. It is calculated by GGOV*RENF.	Authors’ Calculation
FDI	Foreign Direct Investment	Foreign direct investment, net-inflows (% of GDP)	WDI
FDEV	Financial Development	Domestic credit to private sector (% of GDP)	WDI
CO_2_	Carbon Emissions	CO_2_ emissions (metric ton per capita)	WDI
IFR	Inflation	Inflation, consumer prices (annual %)	WDI

Economic growth (EGR) is the dependent variables that is measured by GDP growth (annual %), this variable represents the overall economic performance and health of a country. It is chosen because economic growth is the primary outcome of interest in this study, reflecting how various factors, including energy finance and governance, impact the economic development of BRICS countries.

The study uses two main independent variables FFEF and RENF. FFEF is the proportion of energy finance directed towards fossil fuel projects, expressed as a percentage of total energy finance. It is chosen to evaluate how investments in fossil fuel energy affect economic growth. Fossil fuels have historically been a major energy source, significantly impacting economic activities and growth. On the other hand, RENF represents the share of total final energy consumption that comes from renewable energy sources. It is selected to assess the impact of investments in renewable energy on economic growth. As the world shifts towards sustainable energy, understanding the role of renewable energy finance is crucial for policy-making and economic planning.

Good governance (GGOV) is the main focusing moderating variables in this study. GGOV is measured by government effectiveness (percentile rank), this variable indicates the quality of governance in terms of policy implementation and public service delivery. Good governance is essential for the effective use of resources and sustainable economic development [34]. It is chosen to investigate whether good governance can enhance the positive impacts of energy finance on economic growth. From GGOV, two interaction terms are calculated to explore its moderating effects: First interaction term is GGOV*FFEF that examines how good governance influences the relationship between fossil fuel energy finance and economic growth. Second interaction term is GGOV*RENF that assesses how good governance affects the relationship between renewable energy finance and economic growth.

This study uses four relevant control variables. First, foreign direct investment (FDI) is measured by net inflows as a percentage of GDP, this variable indicates the level of foreign investment in a country. It is chosen as a control variable because foreign direct investment is a crucial driver of economic growth, providing capital, technology transfer, and job creation. Second, financial development (FDEV) is represented by domestic credit to the private sector as a percentage of GDP, this variable reflects the level of financial services and resources available within a country. Financial development is included as it facilitates investment and economic activities, contributing to growth. Third, carbon emission is measured in metric tons per capita, this variable indicates the environmental impact of economic activities. It is selected to account for the environmental dimension of energy finance, especially relevant in the context of sustainable development and the transition to greener energy sources. Fourth, inflation (IFR) is measured by the annual percentage change in consumer prices, this variable reflects the rate of inflation in the economy. Inflation is a critical macroeconomic indicator affecting purchasing power, investment decisions, and economic stability, thus it is included as a control variable.

### Research model

Given Eq (1) depicts a general formulation of the model where economic growth is indicated by fossil fuel energy finance, renewable energy finance, good governance, foreign direct investment, financial development, carbon emissions, and inflation. The specific Eqs (1.1) to (1.6) offer detailed variations that include interaction terms to explore the moderating effects of good governance on the relationships between energy finance and economic growth. The measure of variables is also listed in Table 1.


$$EGR=f(FFEF,RENF,GGOV,FDI,FDEV,CO_{2},IFR)$$ (1)

$${EGR}_{it}=C_{it}+\beta_{1}{FFEF}_{it}+\beta_{2}{GGOV}_{it}+\beta_{3}{CO2}_{it}+\beta_{4}{FDEV}_{it}+\beta_{5}{FDI}_{it}+\beta_{6}{IFR}_{it}+\varepsilon_{it}$$ (1.1)

$${EGR}_{it}=C_{it}+\beta_{1}{FFEF}_{it}+\beta_{2}{GGOV*FFEF}_{it}+\beta_{3}{GGOV}_{it}+\beta_{4}{CO2}_{it}+\beta_{5}{FDEV}_{it}+\beta_{6}{FDI}_{it}+\beta_{7}{IFR}_{it}+\varepsilon_{it}$$ (1.2)

$${EGR}_{it}=C_{it}+\beta_{1}{RENF}_{it}+\beta_{2}{GGOV}_{it}+\beta_{3}{CO2}_{it}+\beta_{4}{FDEV}_{it}+\beta_{5}{FDI}_{it}+\beta_{6}{IFR}_{it}+\varepsilon_{it}$$ (1.3)

$${EGR}_{it}=C_{it}+\beta_{1}{RENF}_{it}+\beta_{2}{GGOV*RENF}_{it}+\beta_{3}{GGOV}_{it}+\beta_{4}{CO2}_{it}+\beta_{5}{FDEV}_{it}+\beta_{6}{FDI}_{it}+\beta_{7}{IFR}_{it}+\varepsilon_{it}$$ (1.4)

$${EGR}_{it}=C_{it}+\beta_{1}{FFEF}_{it}+\beta_{2}{RENF}_{it}+\beta_{3}{GGOV}_{it}+\beta_{4}{CO2}_{it}+\beta_{5}{FDEV}_{it}+\beta_{6}{FDI}_{it}+\beta_{7}{IFR}_{it}+\varepsilon_{it}$$ (1.5)

$${EGR}_{it}=C_{it}+\beta_{1}{FFEF}_{it}+\beta_{2}{RENF}_{it}+\beta_{3}{GGOV*FFEF}_{it}+\beta_{4}{GGOV*RENF}_{it}+\beta_{5}{GGOV}_{it}+\beta_{6}{CO2}_{it}+\beta_{7}{FDEV}_{it}+\beta_{8}{FDI}_{it}+\beta_{9}{IFR}_{it}+\varepsilon_{it}$$ (1.6)

Here, EGR = Economic growth, FFEF = Fossil fuel energy finance, RENF = Renewable energy finance, GGOV = Good governance, CO_2_ = Carbon emissions, FDI = Foreign direct investment, FDEV = Financial development, IFR = Inflation rate, C = Constant, i = Countries, t = Years, β = Coefficients, * = Multiplication that indicates interaction between variables, ε = Error term.

The study adopts a systematic approach, following the methodologies used by Rahman [53], to select the appropriate econometric model for analyzing the impact of energy finance and good governance on economic growth. Initially, a cross-sectional dependency (CSD) test is performed to determine whether cross-sectional dependence exists in the panel data [37]. The presence of CSD indicates that shocks affecting one country might influence others, which is particularly relevant in interconnected economies like those of the BRICS nations.

Subsequently, stationarity tests are conducted to check if the time series data is stationary, meaning that the statistical properties such as mean and variance remain constant over time [54]. Ensuring stationarity is crucial for reliable regression analysis and avoiding spurious results. Following this, a cointegration test is employed to determine if a long-run equilibrium relationship exists among the variables [31]. If the variables are cointegrated, it implies that they move together over time, despite being non-stationary individually [28]. Given the presence of cross-sectional dependency, the stationarity of the variables, and their long-run cointegration, the FMOLS model is selected. FMOLS is well-suited for addressing endogeneity issues and serial correlation, providing unbiased and consistent estimates in the presence of cointegration [37, 38, 55]. To further ensure the robustness of the results, the study employs DOLS. DOLS adjusts for endogeneity and serial correlation by including leads and lags of the differenced independent variables, ensuring more reliable long-run parameter estimates [31].

## Results

### Summary statistics and correlation matrix

Table 2 provides a comprehensive summary of the key variables used in the study, detailing measures of central tendency, dispersion, and distributional characteristics. The mean values represent the average of each variable over the sample period. For instance, the mean CO_2_ emissions is 5.366 metric tons per capita, indicating the typical emissions level in BRICS countries. The average economic growth rate is 4.369% per year, reflecting the general growth trend in these economies. Financial development averages 74.757% of GDP, suggesting a relatively high level of financial services. Foreign direct investment has a mean of 2.274% of GDP, highlighting its role in the economies. Fossil fuel energy finance averages 77.555%, showing the significant reliance on fossil fuels, while renewable energy finance is 24.294%, indicating a growing but still smaller proportion of energy finance. Good governance has a mean percentile rank of 52.918, suggesting moderate governance quality. Inflation averages 6.820%, reflecting varying inflation rates across the sample.

**Table 2:** Summary statistics.

Tests	CO_2_	EGR	FDEV	FDI	FFEF	GGOV	IFR	RENF
Mean	5.366	4.369	74.757	2.274	77.555	52.918	6.820	24.294
Median	5.613	4.444	55.392	2.063	84.903	53.202	5.262	16.746
Maximum	11.640	14.231	182.433	5.368	92.143	83.060	85.746	52.712
Minimum	0.821	-7.800	14.212	0.205	51.319	25.943	-1.401	3.181
Std. Dev.	3.652	4.051	43.228	1.288	13.671	11.433	8.556	17.471
Skewness	0.242	-0.553	0.442	0.344	-0.679	-0.088	6.810	0.261
Kurtosis	1.546	3.554	1.876	2.078	1.873	2.809	61.882	1.449
Jarque-Bera	11.736	7.651	10.222	6.623	15.561	0.338	18263.190	13.393
Probability	0.103	0.122	0.106	0.136	0.200	0.144	0.230	0.201

The median values provide additional insights, indicating the middle value when data is ordered. For CO_2_ emissions, the median is slightly higher at 5.613, suggesting a slight skew towards higher values. The median economic growth rate is 4.444%, close to the mean, indicating a balanced distribution around the average growth rate. The median values for other variables also align closely with their means, except for FDEV and RENF, where the medians are significantly lower, suggesting some skewness.

The maximum and minimum values show the range and variability within the data. CO_2_ emissions range from 0.821 to 11.640 metric tons per capita, reflecting substantial variation across countries and time. Economic growth rates vary widely from -7.800% to 14.231%, highlighting periods of both significant contraction and robust growth. The wide range in FDEV, from 14.212% to 182.433%, indicates diverse levels of financial sector development. The range for FDI, from 0.205% to 5.368%, shows varying levels of foreign investment. FFEF ranges from 51.319% to 92.143%, and RENF from 3.181% to 52.712%, indicating significant shifts in energy finance preferences. GGOV ranges from 25.943 to 83.060, showing varied governance quality, while IFR varies widely from -1.401% to 85.746%, reflecting diverse inflationary environments.

The standard deviation provides a measure of dispersion around the mean. CO_2_ emissions and economic growth have standard deviations of 3.652 and 4.051, respectively, indicating moderate variability. Financial development and renewable energy finance have higher standard deviations, 43.228 and 17.471 respectively, pointing to substantial variation across countries. The standard deviation for FDI is 1.288, indicating relatively lower variability. FFEF, GGOV, and IFR also show considerable variation with standard deviations of 13.671, 11.433, and 8.556 respectively. Skewness and kurtosis further describe the data’s distribution. Skewness values close to zero suggest relatively symmetric distributions, while kurtosis values near 3 indicate distributions similar to the normal distribution. CO_2_ emissions, FDI, and RENF have positive skewness, indicating a longer right tail, while EGR and FFEF are negatively skewed, indicating a longer left tail. Most variables have kurtosis values around 2, suggesting light-tailed distributions, except IFR, which shows a high kurtosis of 61.882, indicating a heavy-tailed distribution.

The Jarque-Bera test assesses the normality of the data by examining the skewness and kurtosis of the sample distribution. The p-values for all variables are above 0.05, indicating that the variables do not significantly deviate from normality and can be considered normally distributed for the purposes of this analysis. This normality assumption supports the validity of using various econometric models in the study.

Table 3 presents the correlation matrix, offering insights into the relationships between key variables used in the study before conducting regression analysis. Each correlation coefficient ranges from -1 to 1, indicating the strength and direction of associations between pairs of variables. CO_2_ exhibit a strong positive correlation of 0.838 with FFEF, suggesting that higher investments in fossil fuel energy are closely linked to increased CO_2_ emissions. Conversely, there is a significant negative correlation of -0.950 between CO_2_ and RENF, indicating that greater investments in renewable energy are associated with lower carbon emissions. These findings underscore the potential impact of energy finance policies on environmental outcomes within the BRICS countries.

**Table 3:** Correlation matrix.

Variables	CO_2_	EGR	FDEV	FDI	FFEF	GGOV	IFR	RENF
CO_2_	1							
EGR	-0.155	1						
FDEV	0.209	0.129	1					
FDI	-0.194	0.207	0.008	1				
FFEF	0.838	-0.121	0.435	-0.220	1			
GGOV	-0.126	0.254	0.631	-0.156	0.160	1		
IFR	0.240	-0.090	-0.386	-0.126	0.126	-0.385	1	
RENF	-0.950	0.041	-0.378	0.158	-0.944	-0.030	-0.180	1

EGR shows modest positive correlations with financial development (FDEV) at 0.129 and foreign direct investment (FDI) at 0.207, suggesting that stronger financial sectors and increased foreign investments may support economic expansion. Additionally, EGR is positively correlated with GGOV at 0.254, indicating that effective governance practices might contribute to higher economic growth rates. Conversely, there is a weak negative correlation between EGR and inflation rate (IFR) at -0.090, implying that higher inflation rates could marginally dampen economic growth. These correlations provide a foundational understanding of the relationships among energy finance, governance, economic growth, and environmental impacts within the BRICS context. They inform the selection of variables and the formulation of regression models to further explore these connections rigorously.

### Findings from CSD test

Table 4 presents the results of cross-sectional dependence (CSD) tests conducted for various variables included in the study, using different statistical methods to assess the interdependencies across countries. The Breusch-Pagan LM, Pesaran scaled LM, Bias-corrected scaled LM, and Pesaran CD tests collectively evaluate whether observations from different countries exhibit significant mutual influences that could bias econometric models [54]. Significant results reveal that variables such as CO_2_ emissions, EGR, FDEV, and FFEF demonstrate notable cross-sectional dependence. This suggests that changes in these variables within one country are likely to influence similar variables in others within the same group. Conversely, variables like IFR and RENF exhibit weaker or insignificant cross-sectional dependence.

**Table 4:** CSD test result.

Variables	Breusch-Pagan LM	Pesaran scaled LM	Bias-corrected scaled LM	Pesaran CD
CO_2_	112.508***	22.921***	22.813**	10.291**
EGR	94.013***	18.786***	18.677**	9.196**
FDEV	113.854***	23.222***	23.114**	9.644**
FDI	17.010	1.567	1.459	0.334
FFEF	61.107***	11.428***	11.319**	-0.696
GGOV	92.956**	18.549**	18.441*	-2.249*
IFR	11.761	0.394	0.285	-0.728
RENF	96.615*	19.368**	19.259*	4.658**

Note: Null hypothesis = No cross-section dependence.

*** = p<0.01

** = p<0.05

* = p<0.10

### Findings from unit root test

The unit root test results are crucial to determine whether variables exhibit trends that can lead to spurious regression results [37]. Table 5 presents the results of unit root tests conducted using second generation methods, CIPS (Cross-Sectionally Augmented IPS) and CADF (Cross-Sectional Augmented Dickey-Fuller), to determine the stationarity properties of the variables in both their levels and first differences [54]. The CIPS test examines whether the variables are stationary in their levels and first differences. A test statistic greater than critical values indicates evidence against the null hypothesis of a unit root (non-stationarity). Variables such as CO_2_, EGR, FDEV, FDI, FFEF, and IFR exhibit significant evidence of stationarity in their first differences, suggesting that these variables become stationary after first differencing. However, in their levels, some variables show mixed results with smaller test statistics, implying they may be non-stationary in their original forms. Similarly, the CADF test assesses the stationarity of variables using an augmented Dickey-Fuller framework. Results indicate significant evidence of stationarity in first differences for CO_2_, EGR, FDEV, FDI, FFEF, IFR, and RENF.

**Table 5:** Unit root rest result.

Variables	CIPS		CADF	
	Level	1st Difference	Level	1st Difference
CO_2_	1.354	-5.528***	7.133	47.592***
EGR	-0.400	-7.960***	13.897	68.630***
FDEV	1.594	-4.510**	5.603	39.338**
FDI	-2.622**	-8.499***	25.752*	75.215***
FFEF	-1.333**	-6.480***	21.244*	56.065***
GGOV	-0.767	-8.647**	11.979	75.435**
IFR	-3.235**	-12.117***	28.364**	109.218***
RENF	0.274	-5.409***	13.803	47.617***

Note

*** = p<0.01

** = p<0.05

* = p<0.10

### Findings from panel cointegration test

Table 6 provides the results of cointegration tests using Pedroni and Kao methods to assess long-run relationships among variables in panel data. The Pedroni tests reveal significant evidence of cointegration within the panel across various dimensions. This suggests that despite short-term fluctuations, there are stable, equilibrium relationships among the variables over time and across countries [31]. Additionally, the Kao test shows a highly significant Augmented Dickey-Fuller statistic (p < 0.01), further supporting the presence of cointegration in the panel data. These findings imply that changes in these variables can mutually influence each other in the long run, underscoring the importance of employing dynamic econometric models to accurately capture these relationships for policy formulation and economic analysis. The Pedroni test was utilized because it accounts for heterogeneity across countries in the panel, allowing for more flexible and nuanced detection of cointegration relationships across various dimensions. The Kao test, on the other hand, was used to complement Pedroni by offering a more stringent confirmation of cointegration, focusing on homogenous panels and providing a robust validation through its Augmented Dickey-Fuller statistic. Together, these tests provide a comprehensive and reliable assessment of long-run relationships among the variables.

**Table 6:** Cointegration test for the panel.

Methods	Tests	Statistic	p-value	Weighted Statistic	p-value
**Pedroni**	***Within-dimension*:**				
	Panel v-Statistic	-1.461	0.028**	-1.043	0.052*
	Panel rho-Statistic	1.563	0.041**	1.329	0.008***
	Panel PP-Statistic	1.392	0.018**	1.083	0.061*
	Panel ADF-Statistic	1.294	0.002***	1.009	0.044**
	***Between-dimension*:**				
	Group rho-Statistic	2.197	0.086*		
	Group PP-Statistic	1.818	0.066*		
	Group ADF-Statistic	1.456	0.027**		
**Kao**	Augmented Dickey–Fuller	-4.403	0.000***		

Note

*** = p<0.01

** = p<0.05

* = p<0.10.

### Findings from long-run estimations

The findings from the FMOLS estimation provide crucial insights into the relationships between energy finance, governance quality, and EGR. FMOLS is particularly robust in panel data settings as it corrects for endogeneity and serial correlation, yielding reliable estimates for long-run relationships [55]. Table 7 highlights significant findings regarding the impact of energy finance types and governance quality on EGR. Firstly, the positive effect of FFEF on EGR aligns with environmental economics literature. Also, the positive impact of RENF on EGR reflects global efforts towards sustainable development and renewable energy adoption. Countries investing more in renewable energy tend to foster economic growth through innovation, job creation, and reduced environmental externalities, thus supporting long-term economic sustainability.

**Table 7:** Long-run estimations using FMOLS.

	M1	M2	M3	M4	M5	M6
FFEF	0.197**	0.305**			0.041	1.018**
	[0.036]	[0.044]			[0.062]	[0.064]
RENF			0.168**	0.267**	0.208**	1.129**
			[0.032]	[0.031]	[0.061]	[0.066]
GGOV*FFEF		0.000				0.107**
		[0.060]				[0.068]
GGOV*RENF				0.005		0.102**
				[0.076]		[0.086]
GGOV	0.092	0.213**	0.036	0.094	0.045	2.202**
	[0.059]	[0.087]	[0.061]	[0.080]	[0.064]	[0.080]
CO_2_	0.796***	0.811***	1.047***	0.961***	1.065***	0.938***
	[0.047]	[0.048]	[0.045]	[0.050]	[0.044]	[0.041]
FDEV	-0.043	-0.063*	-0.044	-0.064*	-0.051**	-0.092*
	[0.029]	[0.032]	[0.032]	[0.032]	[0.030]	[0.031]
FDI	0.454***	0.477***	0.525***	0.533***	0.499***	0.607***
	[0.089]	[0.090]	[0.092]	[0.089]	[0.097]	[0.102]
IFR	0.014	0.071	0.036	0.044	0.056	0.080
	[0.052]	[0.063]	[0.046]	[0.045]	[0.049]	[0.059]
***Diagnostic tests***						
R-squared	0.496	0.508	0.496	0.354	0.504	86.543
Adjusted R-squared	0.448	0.455	0.448	0.285	0.452	79.811
S.E. of regression	3.047	3.027	3.047	3.466	3.037	40.758
Long-run variance	3.506	3.131	3.277	3.041	3.200	2.725

Note

*** = p<0.01

** = p<0.05

* = p<0.10. M1, M2, …., M6 indicate Model 1, Model2, ……, Model 6. Robust standard errors are in the parentheses.

Further, the interaction terms GGOV*FFEF and GGOV*RENF indicate how good governance moderates these relationships in Table 7. Good governance practices, such as effective regulatory frameworks, transparency, and accountability, can amplify the positive effects of renewable energy investments on economic growth, as they ensure efficient allocation of resources and support technological advancements in clean energy sectors. Similarly, good governance can mitigate the negative impact of fossil fuel energy finance on economic growth by promoting policies that encourage cleaner technologies, environmental protection measures, and sustainable resource management practices. Thus, these moderating effects underscore the importance of governance quality in shaping the outcomes of energy finance policies on economic growth, emphasizing the need for supportive institutional frameworks to maximize the benefits of renewable energy investments while minimizing the drawbacks of fossil fuel dependence.

The findings regarding the control variables provide nuanced insights into their respective impacts on EGR as observed in the study. Firstly, the positive relationship between CO_2_ and EGR suggests that countries experiencing higher levels of CO_2_ emissions tend to also exhibit greater economic growth. This relationship could be attributed to industrialization and economic activities that contribute significantly to GDP but also emit substantial amounts of CO_2_, such as manufacturing and energy production sectors. However, this positive association underscores potential trade-offs between economic development and environmental sustainability, highlighting the challenge of balancing economic growth with environmental stewardship. Secondly, the negative impact of FDEV on EGR indicates that countries with higher levels of domestic credit to the private sector relative to GDP tend to experience slower economic growth. This finding may reflect issues such as credit misallocation, financial market inefficiencies, or excessive debt burdens, which could constrain entrepreneurial activity, investment, and overall economic productivity over time.

Conversely, the positive relationship between FDI and EGR suggests that higher levels of net inflows of foreign direct investment contribute positively to economic growth. FDI often brings in capital, technology, managerial expertise, and access to new markets, stimulating economic activity, job creation, and infrastructure development in host countries. This finding underscores the importance of attracting and effectively utilizing foreign investment to enhance economic growth prospects. Lastly, the lack of significant relationship between inflation (IFR) and EGR indicates that changes in consumer price levels do not exert a statistically significant impact on economic growth in the studied context. This finding suggests that inflationary pressures, within the observed range, may not substantially hinder or stimulate overall economic performance. Overall, these insights underscore the multifaceted nature of factors influencing economic growth, emphasizing the need for balanced policy frameworks that consider both economic development goals and environmental sustainability concerns.

### Robustness check with DOLS

The study employs DOLS to ensure the robustness and reliability of its findings regarding the relationships between energy finance, good governance, and EGR. Table 8 presents the results from DOLS. The similar results across different methods, including OLS, suggest consistency and stability in the estimated relationships between variables [56]. This consistency reinforces the initial findings derived from the primary model (Eq 1.6), confirming the direction and significance of the impacts of FFEF, RENF, and good governance on economic growth.

**Table 8:** Impact on economic growth using DOLS model.

	M1	M2	M3	M4	M5	M6
FFEF	0.276**	0.264**			0.464***	1.704***
	[0.051]	[0.158]			[0.089]	[0.572]
RENF			0.235*	0.353*	0.329**	1.448**
			[0.080]	[0.144]	[0.130]	[0.515]
GGOV*FFEF		0.000				0.023*
		[0.004]				[0.011]
GGOV*RENF				0.003		0.022*
				[0.003]		[0.010]
GGOV	0.070**	0.048	0.097**	0.023	0.070**	2.381**
	[0.041]	[0.282]	[0.044]	[0.086]	[0.040]	[1.051]
CO_2_	-0.899**	-0.902**	-1.110**	-0.973**	-0.011	0.000
	[0.182]	[0.186]	[0.368]	[0.393]	[0.393]	[0.393]
FDEV	-0.022*	-0.022	-0.020	-0.008	-0.012	-0.021
	[0.011]	[0.015]	[0.013]	[0.017]	[0.012]	[0.016]
FDI	0.910**	0.913**	0.690**	0.693**	1.144**	1.212**
	[0.263]	[0.266]	[0.287]	[0.287]	[0.273]	[0.272]
IFR	0.005	0.006	0.009	0.006	0.021	0.033
	[0.043]	[0.044]	[0.047]	[0.048]	[0.043]	[0.043]
Constant	-16.420**	-15.429	10.769**	11.808**	-45.161**	-166.316**
	[3.288]	[12.985]	[5.398]	[5.498]	[11.813]	[56.095]
***Diagnostic tests***						
R-squared	0.318	0.318	0.200	0.207	0.355	0.383
Adjusted R-squared	0.282	0.276	0.157	0.157	0.315	0.333
S.E. of regression	3.433	3.448	3.720	3.720	3.354	3.309
F-statistic	8.790***	7.469***	4.694***	4.165***	8.807***	7.596***
Long-run variance	3.203	2.410	3.431	3.129	3.121	3.116

Note

*** = p<0.01

** = p<0.05

* = p<0.10. M1, M2, …., M6 indicate Model 1, Model2, ……, Model 6. Robust standard errors are in the parentheses.

## Discussions, theoretical implications, and policy implications

### Discussions

The results of this study offer crucial insights into the relationship between energy finance, governance, and economic growth in BRICS nations. Aligning with the first research question, *“How does fossil fuel energy finance affect economic growth in BRICS nations*?*”* the study confirms that FFEF does positively influence economic growth, supporting Hypothesis 1. BRICS countries, heavily reliant on fossil fuels for their energy needs, have seen robust economic growth largely driven by investments in fossil fuel sectors such as oil, gas, and coal. This positive relationship stems from the significant contributions of fossil fuel industries to industrial productivity, job creation, and overall economic activity in these nations. However, despite the positive short-term effects, the findings also raise concerns about the long-term sustainability of relying on fossil fuel finance due to environmental risks and the potential negative consequences for future economic stability. This aligns with prior research, which emphasizes the economic benefits of fossil fuels but acknowledges their negative externalities [3, 4, 15, 16].

In addressing the second research question, *“How does renewable energy finance impact economic growth in BRICS nations*?*”* the study finds that RENF also positively impacts economic growth, as posited in Hypothesis 2. The results show that investments in renewable energy sources like solar, wind, and hydropower are contributing to economic growth in these emerging economies. By generating new industries, reducing dependence on energy imports, and promoting innovation, RENF enhances economic development. The findings highlight the increasing role of renewables in the energy mix of BRICS nations, especially in countries like China and India, where large-scale renewable energy projects have stimulated economic growth while also addressing environmental concerns. This result is crucial, as it underscores the potential for BRICS nations to transition from fossil fuel dependence to renewable energy sources without sacrificing economic progress.

The study’s third research question, *“What role does governance play in moderating the effects of FFEF and RENF on economic growth*?*”* is pivotal in understanding how institutional frameworks influence the energy-finance-growth nexus. The results confirm Hypothesis 3, showing that good governance directly improves economic growth in BRICS nations. Countries with stronger governance structures—characterized by transparency, accountability, and effective regulatory systems—tend to experience higher levels of economic growth. These governance systems ensure that resources are allocated efficiently and that energy investments, whether in fossil fuels or renewables, are managed in a way that promotes both economic and environmental objectives. This finding aligns with existing governance theories, which emphasize the importance of robust institutions in fostering long-term economic development [18, 25, 27, 28].

Furthermore, the study confirms Hypothesis 4, demonstrating that good governance strengthens the relationship between energy finance and economic growth. In BRICS nations with stronger governance, the negative effects of FFEF, such as environmental degradation and resource mismanagement, are mitigated through effective regulatory oversight. Strong governance helps in reducing corruption and inefficiencies, ensuring that fossil fuel investments contribute positively to the economy without exacerbating environmental damage. Additionally, good governance amplifies the positive effects of RENF on economic growth by streamlining regulatory processes, ensuring accountability, and promoting policies that favor the expansion of renewable energy sectors. This not only attracts foreign direct investment (FDI) into the renewable energy space but also ensures that environmental goals are met alongside economic growth.

### Theoretical implications

The findings of this study have significant theoretical implications for understanding the dynamics of energy finance, governance, and economic growth, particularly in the context of emerging economies like the BRICS nations. Firstly, the confirmation of the positive impacts of both FFEF and RENF on economic growth contributes to the ongoing discourse on energy economics by highlighting the dual role that different energy sources can play in driving economic development. Moreover, the study reinforces governance theory by illustrating how strong institutional frameworks can serve as critical moderators in the relationship between energy finance and economic growth. By demonstrating that effective governance enhances the positive effects of RENF while mitigating the negative consequences of FFEF, this research underscores the need for robust governance structures to ensure that energy investments align with both economic and environmental objectives. These insights not only refine existing theories related to energy finance and economic growth but also emphasize the importance of integrating governance considerations into the analysis of energy investment strategies in emerging economies. Ultimately, this study enriches the theoretical landscape by proposing a more nuanced understanding of how governance can shape the sustainability and effectiveness of energy finance in promoting long-term economic growth.

### Policy recommendations

Based on the study’s findings, several targeted policy recommendations can enhance energy finance strategies and promote economic growth in BRICS nations while ensuring environmental sustainability. Firstly, BRICS countries should develop carefully designed energy financing policies that facilitate the transition to renewable energy sources while maintaining steady economic growth. Policymakers should prioritize renewable energy investments by offering incentives such as tax breaks and subsidies, which can help remove cost barriers associated with energy transitions. Strengthening governance frameworks is crucial; improving transparency, reducing corruption, and enhancing institutional effectiveness will boost investor confidence and ensure that energy finance contributes positively to economic development. Furthermore, good governance acts as a moderating factor, mitigating risks associated with mismanagement and ensuring that the benefits of energy finance are fully realized.

BRICS nations should also foster public-private partnerships to leverage private investment and drive innovation in renewable energy projects. This collaboration can accelerate infrastructure development and expand energy access, improving living conditions and boosting local economies. Additionally, investing in capacity-building programs will ensure that the workforce possesses the necessary skills to support the renewable energy sector. Establishing clear environmental regulations for fossil fuel projects is essential to minimize negative impacts, while effective enforcement mechanisms are needed to hold companies accountable. Finally, fostering international cooperation will allow BRICS nations to share best practices, technologies, and financial resources for energy transition. By implementing these strategic actions, BRICS nations can effectively navigate the complexities of energy finance, promote sustainable economic growth, and ensure that governance frameworks enhance the overall success of energy initiatives.

## Conclusions

This study provides a comprehensive examination of the relationship between energy finance, governance, and economic growth in BRICS nations, revealing critical insights that contribute to both academic discourse and policy formulation. The findings confirm that both FFEF and RENF positively impact economic growth, albeit through different mechanisms. While FFEF has historically driven economic activity in these nations, the study underscores the importance of transitioning towards RENF to ensure sustainable development that aligns with global environmental goals. Moreover, the role of governance emerges as a pivotal factor in moderating the relationship between energy finance and economic growth. Strong governance frameworks enhance the positive effects of RENF while mitigating the negative consequences of FFEF, ensuring that energy investments are managed effectively and transparently. This interplay highlights the necessity for BRICS nations to prioritize good governance as they navigate their energy finance strategies.

The study’s implications extend beyond theoretical contributions; it offers actionable policy recommendations aimed at fostering sustainable economic growth. By prioritizing renewable energy investments, strengthening governance structures, and fostering public-private partnerships, BRICS countries can effectively address the challenges of energy transitions while enhancing living conditions and boosting local economies. Ultimately, this research emphasizes the need for a balanced approach to energy finance that not only fuels economic growth but also safeguards environmental sustainability. As BRICS nations continue to play a significant role in the global economy, their ability to implement these strategies will be critical in shaping a sustainable future, both for their populations and for the broader international community.

Despite its contributions, this study has limitations that warrant consideration. Firstly, its focus exclusively on BRICS countries restricts the generalizability of findings to other regions or contexts with different economic structures or governance frameworks. Secondly, the study primarily examines direct relationships between energy finance, good governance, and economic growth, overlooking potential mediating factors that could influence these relationships, such as technological advancements, energy efficiency measures, or specific sectoral impacts. Additionally, certain variables that are known to affect economic growth and environmental outcomes, such as education levels, technological innovation, and demographic factors, were not included in the analysis. Future research could benefit from incorporating these variables to provide a more comprehensive understanding of the complex dynamics at play in energy-related economic development strategies. Addressing these limitations would enhance the study’s applicability and robustness, offering deeper insights into effective policy interventions for sustainable economic growth.

## Supporting information

S1 File (XLSX)
